# Effects of opening and closing six-qi acupuncture as adjuvant therapy for sleep disorders of elderly

**DOI:** 10.1097/MD.0000000000043841

**Published:** 2025-08-08

**Authors:** Ziping Luo, Wenkun Luo, Qun Yi

**Affiliations:** a Department of Traditional Chinese Medicine, Hengyang No.1 People’s Hospital, Hengyang, China; b School of Chemistry and Pharmaceutical Engineering, Changsha University of Science and Technology, Changsha, China.

**Keywords:** adjuvant therapy, elderly, opening and closing six-qi acupuncture (OCSQA), sleep disorder

## Abstract

Sleep disorder is essentially a huge threat to both physiological and psychological health of human being, especially elderly, and population aging has exacerbated the situation. Hypnotics are effective therapy for sleep disorder, while side-effects are frequently observed after long-term medication of hypnotics. The effects of opening and closing six-qi acupuncture (OCSQA) as an adjuvant therapy of hypnotics (i.e., Estazolam) treatment for sleep disorder of elderly were explored based on the Pittsburgh sleep quality index scores, the traditional Chinese medicine syndrome scores, the serum levels of immunoglobulins (IgG, IgA, and IgM) and the overall efficacy. Hypnotics could effectively relieve sleep disorder of elderly; OCSQA had positive impacts on the relief of sleep disorder by hypnotics, and can relieve side-effects (e.g., anxiety, ataxia) induced by medication of hypnotics. Additionally, no significant adverse events were reported. OCSQA is an effective and safe adjuvant therapy for sleep disorder of elderly due to therapeutic benefits on treatment with hypnotics.

## 1. Introduction

As one of the key functions for most living species, sleep plays an important role in our daily life. Indeed, approximately 30% of human life is spent on sleep. Sleep is of great significance to immunity, body development and memory shaping, helping people to stay focused, maintain good mood, and make rational decisions.^[1,2]^ Due to the importance of sleep, sleep disorder, which refer to various functional impairments present during falling asleep and awakening, can be a huge threat to both physiological and psychological health of human being.^[3–5]^ It has been demonstrated that poor or insufficient sleep caused by sleep disorder is associated with endocrine, metabolic, cortical and neurological dysfunctions, and eventually diseases (e.g., coronary atherosclerotic heart disease, depression). In modern society, fast-paced life, high-fat diet and intensive work tend to make people exhausted, and sleep disorder is essentially devasting, especially when combined with other diseases (e.g., obesity, cardiovascular diseases, diabetes). In other words, life quality of a modern citizen is highly dependent on the sleep quality.

Population aging has been a severe issue all over the world, especially in China. Currently, 20.7% (297 million) of China’s population are elderly citizens aged above 60 years and 35.8 million of them are aged above 80 years.^[6]^ Therefore, health of the elderly population has attracted great attention as diseases of the elderly community can be financial and psychological burdens to both the patients and their families. Due to drastic yet inevitable degradation of body functions, the sleep quality of elderly people is significantly lower than that of their young peers, and most elderly people are facing sleep disorder, which significantly affects their immunity and eventually causes various diseases and physiological disorders. Previous studies have demonstrated that sleep disorder such as insomnia is closely related to the mortality of elderly population,^[7,8]^ especially those aged above 80 years.

Currently, treatment of sleep disorder relies primarily on hypnotics, including zolpidem (Ambien),^[9]^ benzodiazepines,^[10]^ antipsychotics and zaleplon (Sonata).^[11]^ Despite that hypnotics are effective and efficient therapy for sleep disorder, the patients may be exposed to various side-effects after long-term medication, including anxiety, ataxia, hallucinations, and drug dependence. To make it worse, administration of hypnotics has been demonstrated to be associated with fatal events such as heart attack and stroke.^[12]^ Additionally, hypnotics are strictly controlled as they could contribute to drug abuse and suicide, and administration of hypnotics requires frequent visit to clinics and/or hospitals.^[13]^ Therefore, adjuvant therapies that can reduce the dosage of hypnotics used are urgently needed, especially for elderly patients with underlying diseases.

As a key tool in traditional Chinese medicine, acupuncture has been applied for treatment and recovery in China for over 2000 years.^[14–16]^ It involves stimulation of acupoints by needle insertion to induce the *Deqi* sensation for therapeutic purposes. Acupuncture can be divided into manual acupuncture, acupressure, electroacupuncture, cupping and moxibustion, and they work under different scenarios. Nowadays, acupuncture plays a key role in complementary and alternative medicine. Previous studies have demonstrated that acupuncture can work directly on the nervous system.^[17–19]^ For instance, Aroxa *et al* explored the efficacy of acupuncture as adjuvant therapy for sleep disorders among patients with Parkinson Disease, and found that acupuncture led to significantly improved quality of nocturnal sleep of the subjects.^[20]^ Yue *et al* summarized most recent cases from 5 regions highlighting the translational potential of complementary and alternative therapies in clinical application.^[21]^ Sheng *et al* reported that acupuncture serves as a feasible adjunctive therapy to enhance sleep quality of patients with Parkinson Disease.^[22]^

**Figure 1.**
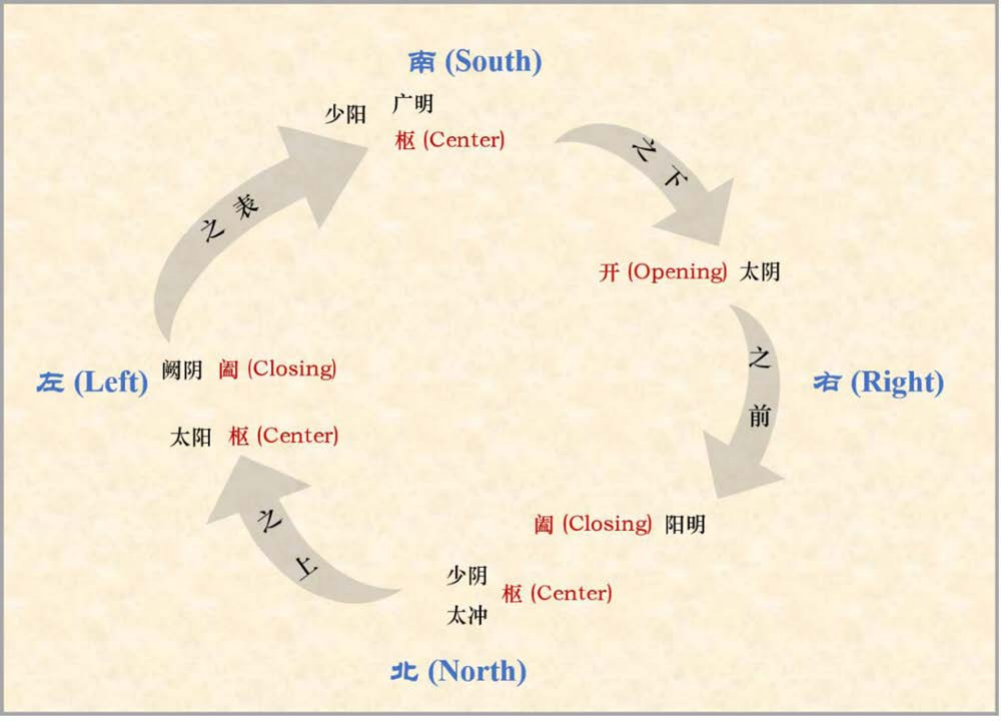
Schematic of “Three Yin, Three Yang” 6 meridians used in OCSQA. OCSQA = opening and closing six-qi acupuncture.

Among various acupuncture reported to date, opening and closing six-qi acupuncture (OCSQA, see Fig. 1) is a representative one. As a unique approach based on the “opening-closing-center” theory and the “six meridian” theory, OCSQA has been widely employed as adjuvant therapy for neurologic and neurogenerative diseases (e.g., Alzheimer disease, Parkinson disease) in virtue of good therapeutic efficacy. For instance, Tao *et al* reported that OCSQA combined with felodipine showed high therapeutic efficacy for hypertension and application of OCSQA led to further improved efficacy compared with felodipine alone. Nevertheless, few studies of the efficacy of OCSQA as adjuvant therapy for sleep disorder have been reported to date, and none of them focused on the elderly population, which is more susceptible to insomnia and sleep disorder.

In this study, the effects of OCSQA as an adjuvant therapy of hypnotics (i.e., Estazolam) treatment for sleep disorder of elderly were explored based on a series of indicators. Specifically, the Pittsburgh sleep quality index (PSQI) scores, the traditional Chinese medicine syndrome (TCMS) scores, the serum levels of immunoglobulins (IgG, IgA and IgM) and the overall efficacy of the hypnotics group, the hypnotics + OCSQA group and the placebo group were investigated. Also, adverse events were recorded to facilitate safety assessment of OCSQA.

## 2. Materials and methods

### 2.1. Participants

A total of 49 elderly patients with sleep disorder diagnosed by neurologists in Hengyang No. 1 People’s Hospital based on the Chinese Guideline for Diagnosis and Treatment of Insomnia (2023)^[23]^ and international classification of sleep disorders – third edition (ICSD-3)^[24]^ were recruited, while 13 of them were excluded based on the inclusion and exclusion criteria (see Fig. S1, Supplemental Digital Content, https://links.lww.com/MD/P631). The demographic data of the 36 participants enrolled in this study are summarized in Table S1, Supplemental Digital Content, https://links.lww.com/MD/P632. Specifically, the 36 participants comprised 20 male and 16 female; their ages were 61 to 76 years, with an average of 68.1 years; most of them had fundamental diseases, including diabetes, hypertension, coronary heart disease, Alzheimer disease, chronic obstructive pulmonary disease, and asthma.

### 2.2. Eligibility criteria

Participants met the following inclusion criteria were enrolled: the participant was aged 60 to 80 years; the participant had been suffering from sleep disorder affecting their daily life for no less than 4 weeks; the participant had a score of the PSQI ≥ 11; the participant adhere to acupuncture treatment protocols and scale assessments required in this study; no treatment and therapy other than the proposed therapy was received 1 month before the study and during the study or the follow-up period; demographic and clinical data, including age, gender, disease duration, and other pertinent medical information, is available and accessible.

Participants were excluded from the study if they meet any of the following criteria: the participant was aged below 60 years or above 80 years; the participant was diagnosed with other severe neurological disorders that may affect the efficacy of the proposed therapy or the outcome of the assessments; the participant received treatment in other hospitals/institutions during the study; the participant showed low compliance that may affect the efficacy of the proposed therapy; the participant had contraindications (e.g., allergies) to acupuncture treatment; the participant quitted the study for personal reasons.

### 2.3. Intervention

The participants were randomly (by simple randomization) divided into the hypnotics group (Group A, n = 12), the hypnotics + OCSQA group (Group B, n = 12) and the control group (Group C, n = 12), and the intervention lasted for 10 weeks. For the hypnotics group, administration of Estazolam (Shanghai Pharmaceuticals Holdings Co., Ltd., 0.5 mg per time, once a day) was applied; for the hypnotics + OCSQA group, both administration of Estazolam (0.5 mg per time, once a day) and OCSQA (twice a week) were applied; for the control group, placebo was applied. Herein, the OCSQA was applied as follows: the patient sat facing south, and the physician stood behind the patient; the physician drew a circle with a diameter of 6 to 8 cm centered on the acupoint of *Baihui*, and identified sensitive sites near the acupoints of *Shaoyang* and *Yangming* areas on top of the head; a 0.25 mm × 40 mm needle was used for acupuncture in a clockwise direction along the circle; the needle was left untouched for 20 minutes at each site. The acupuncture was executed by the same physician team.

### 2.4. Clinical assessment

The PSQI scores, the TCMS scores, the serum levels of immunoglobulins (IgG, IgA and IgM) and the overall efficacy of the 3 groups were determined.

#### 2.4.1. PSQI

The PSQI is a self-report questionnaire that has been widely used for assessment of sleep quality.^[25,26]^ The scores of all participants were determined before, during and after intervention. The PSQI questionnaire used in this study comprised 7 sections, including subjective sleep quality, sleep latency, sleep duration, sleep efficiency, sleep disturbance, use of sleep medication, and daytime dysfunction. The PSQI score (range = 0–21) is a sum of the scores (range = 0–3) of all sections, and it is negatively related to the sleep quality.

#### 2.4.2. TCMS

The TCMS is a profile of symptoms and signs indicating human homeostasis and quantifying severity of syndromes. It enables objective and universal evaluation of traditional Chinese medicine symptoms in both clinical practice and studies by grading various symptoms and assigning scores.^[27,28]^ Hence, TCMS provides references and guidance for acupuncture. TCMS scores of all participants were determined before, during and after intervention. The TCMS questionnaire used in this study was developed according to Specification of Diagnosis and Therapeutic Effect Evaluation of Diseases and Syndromes in Traditional Chinese Medicine.^[29]^ Specifically, it comprised 4 sections, and the TCMS score is a sum of the scores of all questions. The TCMS score is negatively related to the overall health of the subject.

#### 2.4.3. Serum levels of immunoglobulins

As glycoprotein molecules produced by white blood cells, immunoglobulins are essentially antibodies present in serum and blood, and they can eliminate viruses by specifically binding to them. Therefore, immunoglobulins can serve as effective indicators of various clinical diseases, including infection, immunodeficiency, and autoimmune diseases.^[30,31]^ It has been demonstrated that immunoglobulin levels varied significantly in cases of sleep disorder and deficiency.^[32,33]^ Hence, serum levels of immunoglobulins (IgG, IgA and IgM) of all participants were measured using a biochemical analyzer (Indiko^TM^ Clinical Chemistry Analyzer, Thermo Fisher Scientific) before, during and after intervention.

#### 2.4.4. Overall efficacy

The overall efficacy was determined according to the TCM clinical guidelines of insomnia research group (WHO/WPO)^[34]^: significant efficacy: no insomnia symptoms are observed, sleep duration exceeds 6 hours, no daytime dysfunctions are detected, and no diseases caused by external factors are reported over the past month; good efficacy: insomnia symptoms are significantly relieved, sleep duration is 3 to 6 hours, daytime dysfunctions are barely detected, and few diseases caused by external factors are reported; reasonable efficacy: insomnia symptoms are relieved, sleep duration increases but remains <3 hours, the frequency of daytime dysfunctions is reduced, and diseases caused by external factors are easily recovered; negligible efficacy: insomnia symptoms are barely relieved and the frequency of daytime dysfunctions is barely reduced.

### 2.5. Statistical analysis

Statistical analysis was conducted using SPSS (version 22.0, Chicago). All data were expressed as mean ± standard deviation ($\bar{x}\pm s$). Paired sample t-test and independent sample *t*-test were employed for intra- and inter-group comparisons, respectively. Also, *P* <.05 denotes statistically significant differences.

### 2.6. Ethics statement

This study was a randomized controlled clinical trial based on the CONSORT 2010 checklist. All participants had signed informed consent and were free to quit anytime during the study. Protocols in this study were approved by the Ethics Committee of the Hengyang No.1 People’s Hospital (HengYi Lunli 2024015).

## 3. Results

### 3.1. General information

**Figure 2.**
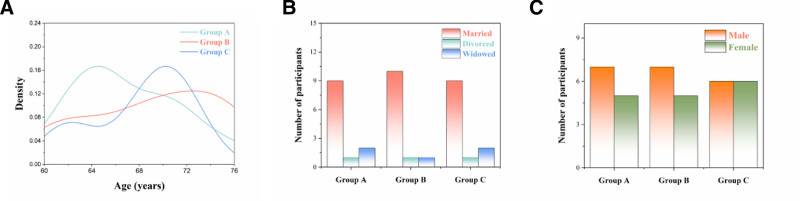
(A) Distributions of participant ages of Groups A, B and C; (B) distributions of marital status of participants in Groups A, B and C; (C) distributions of gender of participants in Groups A, B and C.

The general information of the 3 groups, including gender, age, marital status and fundamental diseases, had no statistically significant differences (*P* >.05), as shown in Figure 2.

### 3.2. PSQI scores

**Figure 3.**
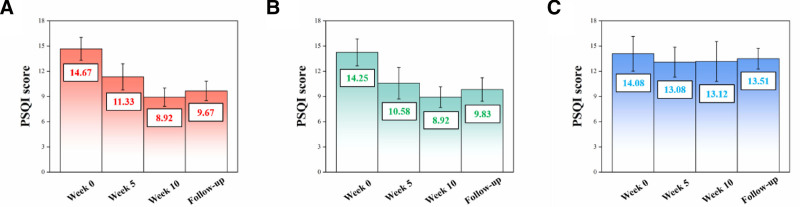
Pittsburgh sleep quality index scores of (A) Group A, (B) Group B and (C) Group C at different stages of intervention (week 0, week 5, week 10 and follow-up).

Table 1 and Figure 3 illustrate the PSQI scores of Group A, Group B and Group C at different stages intervention, including before intervention (week 0), during intervention (week 5), and after intervention (week 10 and follow-up, specifically 3 months after the last intervention activity). As observed, administration of hypnotics could significantly reduce anxiety and other negative emotions (Fig. 3A vs C), which is consistent with previous studies.^[35]^ Meanwhile, the application of OCSQA had positive effects on relief of sleep disorder, especially in short-term (before Week 5) (Fig. 3B vs A). Additionally, slight withdrawal symptoms were found in both Groups A and B, as indicated by increases in the PSQI of participants after intervention compared with during intervention, and the increase in the PSQI was larger in Group B than in Group A, further confirming the positive effects of OCSQA on relief of sleep disorder.

**Table 1:** **Pittsburgh sleep quality index scores of (A) Group A, (B) Group B and (C) Group C before intervention (week 0), during intervention (week 5), and after intervention (week 10 and follow-up)** ($\bar{x}\pm \;s$**).**

Group	Number of cases	Week 0	Week 5	Week 10	Follow-up
A	n = 12	14.67 ± 1.37	11.33 ± 1.56	8.92 ± 1.08	9.67 ± 1.15
B	n = 12	14.25 ± 1.60	10.58 ± 1.88	8.92 ± 1.24	9.83 ± 1.40
C	n = 12	14.08 ± 2.06	13.08 ± 1.78	13.12 ± 2.37	13.51 ± 1.24

### 3.3. TCMS scores

**Figure 4.**
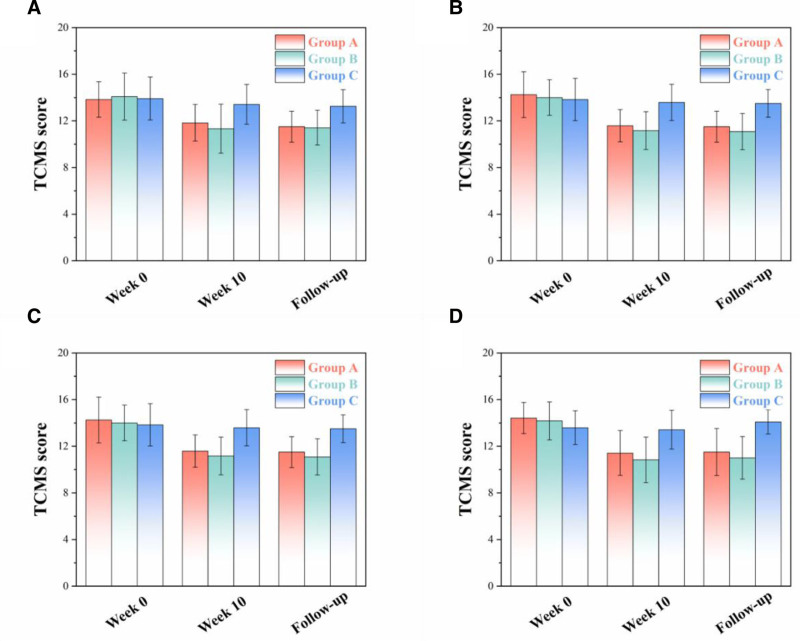
Scores of different TCMS sections ([A] frequent dreaming, [B] anxiety, [C] night sweating, [D] susceptibility to influenza) of different groups before (week 0) and after intervention (week 10 and follow-up). TCMS = traditional Chinese medicine syndrome.

Figure 4 shows scores of different TCMS sections (frequent dreaming, anxiety, night sweating, susceptibility to influenza) of different groups before intervention (week 0), after intervention (week 10) and during follow-up (3 months after the last intervention activity).

#### 3.3.1. Frequent dreaming

It has been demonstrated that frequent dreaming is closely related to insomnia.^[36]^ Hence, it is involved as a section of TCMS in this study. According to Table 2 and Figure 4A, the score of frequent dreaming of Group A decreased from 13.83 to 11.83 after intervention. More importantly, the score of frequent dreaming of Group B decreased from 14.08 to 11.33 after intervention. Additionally, the score of frequent dreaming of Group C varied slightly (from 13.91 to 13.41) after intervention.

**Table 2:** **Scores of frequent dreaming of different groups before (week 0) and after intervention (week 10 and follow-up)** ($\bar{x}\pm \;s$**).**

Group	Number of cases	Week 0	Week 10	Follow-up
A	n = 12	13.83 ± 1.52	11.83 ± 1.57	11.50 ± 1.32
B	n = 12	14.08 ± 2.02	11.33 ± 2.09	11.41 ± 1.50
C	n = 12	13.92 ± 1.85	13.42 ± 1.71	13.25 ± 1.42

#### 3.3.2. Anxiety

Anxiety is essentially a key indicator in traditional Chinese medicine and was involved as a section of TCMS in this study. According to Table 3 and Figure 4B, the score of frequent dreaming of Group A decreased from 13.91 to 11.50. More importantly, the score of anxiety of Group B decreased from 14.33 to 10.83 after intervention. Additionally, the score of anxiety of Group C showed a change <2% before and after intervention.

**Table 3:** **Scores of anxiety of different groups before (week 0) and after intervention (week 10 and follow-up)** ($\bar{x}\pm \;s$**).**

Group	Number of cases	Week 0	Week 10	Follow-up
A	n = 12	13.92 ± 1.61	11.50 ± 1.93	11.58 ± 1.55
B	n = 12	14.33 ± 1.84	10.83 ± 1.40	10.67 ± 1.31
C	n = 12	14.08 ± 1.98	13.92 ± 1.66	13.75 ± 2.00

#### 3.3.3. Night sweating

In traditional Chinese medicine, night sweating is an effective indicator of low sleep quality, if not sleep disorder. In this study, night sweating was involved as a section of TCMS. According to Table 4 and Figure 4C, the score of night sweating of Group A decreased from 14.25 to 11.58. More importantly, the score of night sweating of Group B decreased from 14.00 to 11.17 after intervention. Additionally, the score of anxiety of Group C showed a change <3% before and after intervention.

**Table 4:** **Scores of night sweating of different groups before (week 0) and after intervention (week 10 and follow-up)** ($\bar{x}\pm \;s$**).**

Group	Number of cases	Week 0	Week 10	Follow-up
A	n = 12	14.25 ± 1.96	11.58 ± 1.38	11.50 ± 1.32
B	n = 12	14.00 ± 1.53	11.17 ± 1.62	11.08 ± 1.55
C	n = 12	13.83 ± 1.82	13.58 ± 1.55	13.50 ± 1.19

#### 3.3.4. Susceptibility to influenza

Susceptibility to influenza is regarded as an indicator of sleep quality as it directly reflects the immunity condition of an individual, which is closely related to sleep quality. Hence, susceptibility to influenza was involved as a section of TCMS. According to Table 5 and Figure 4D, the score of susceptibility to influenza of Group A decreased from 14.42 to 11.42. More importantly, the score of susceptibility to influenza of Group B decreased from 14.17 to 10.83 after intervention. Additionally, the score of susceptibility to influenza of Group C showed a change <2% before and after intervention.

**Table 5:** **Scores of susceptibility to influenza of different groups before (week 0) and after intervention (week 10 and follow-up)** ($\bar{x}\pm \;s$**).**

Group	Number of cases	Week 0	Week 10	Follow-up
A	n = 12	14.42 ± 1.34	11.42 ± 1.92	11.50 ± 2.02
B	n = 12	14.17 ± 1.62	10.83 ± 1.95	11.00 ± 1.83
C	n = 12	13.58 ± 1.44	13.42 ± 1.66	14.08 ± 1.04

### 3.4. Serum levels of immunoglobulins

**Figure 5.**
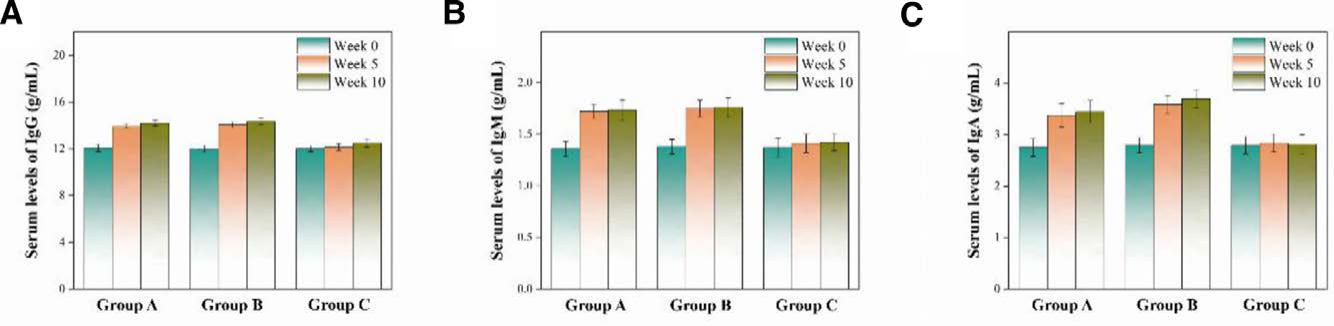
Serum levels of (A) IgG, (B) IgA and (C) IgM of Groups A, B and C before (week 0), during (week 5) and after (week 10) intervention ($\bar{x}\pm s$, g/mL).

Figure 4 illustrates the serum levels of immunoglobulins, including IgG, IgA, and IgM, of Groups A, B and C before (week 0), during (week 5) and after (week 10) intervention. According to Table 6 and Figure 5A, the serum levels of IgG increased significantly in Groups A and B as the intervention progressed, but stayed constant in Group C despite a slight increase after intervention. Meanwhile, the increase was higher in Group B than in Group C. According to Table 7 and Figure 5B, the serum levels of IgA increased in Groups A and B during and after intervention, but stayed constant in Group C. The differences of serum levels of IgA between Group A and Group B were detectable. According to Table 8 and Figure 5C, the serum levels of IgM increased in Groups A and B during and after intervention, but showed negligible changes in Group C. The serum levels of IgM between Group A and Group B were consistent, with non-detectable differences.

**Table 6:** **Serum levels of IgG of different groups before (week 0), during (week 5) and after (week 10) intervention** ($\bar{x}\pm \;s$**, g/mL).**

Group	Number of cases	Week 0	Week 5	Week 10
A	n = 12	12.07 ± 0.31	13.92 ± 0.19	14.18 ± 0.24
B	n = 12	11.99 ± 0.29	14.06 ± 0.24	14.32 ± 0.29
C	n = 12	12.02 ± 0.27	12.14 ± 0.31	12.49 ± 0.35

IgG = immunoglobulin G.

**Table 7:** **Serum levels of IgA of different groups before (week 0), during (week 5) and after (week 10) intervention** ($\bar{x}\pm \;s$**, g/mL).**

Group	Number of cases	Week 0	Week 5	Week 10
A	n = 12	2.75 ± 0.17	3.37 ± 0.22	3.44 ± 0.22
B	n = 12	2.79 ± 0.15	3.58 ± 0.18	3.67 ± 0.19
C	n = 12	2.79 ± 0.17	2.83 ± 0.17	2.81 ± 0.18

IgA = immunoglobulin A.

**Table 8:** **Serum levels of IgM of different groups before (week 0), during (week 5) and after (week 10) intervention** ($\bar{x}\pm \;s$**, g/mL).**

Group	Number of cases	Week 0	Week 5	Week 10
A	n = 12	1.36 ± 0.07	1.72 ± 0.07	1.73 ± 0.10
B	n = 12	1.38 ± 0.07	1.75 ± 0.08	1.76 ± 0.09
C	n = 12	1.37 ± 0.09	1.41 ± 0.09	1.42 ± 0.07

IgM = immunoglobulin M.

### 3.5. Overall efficacy

**Figure 6.**
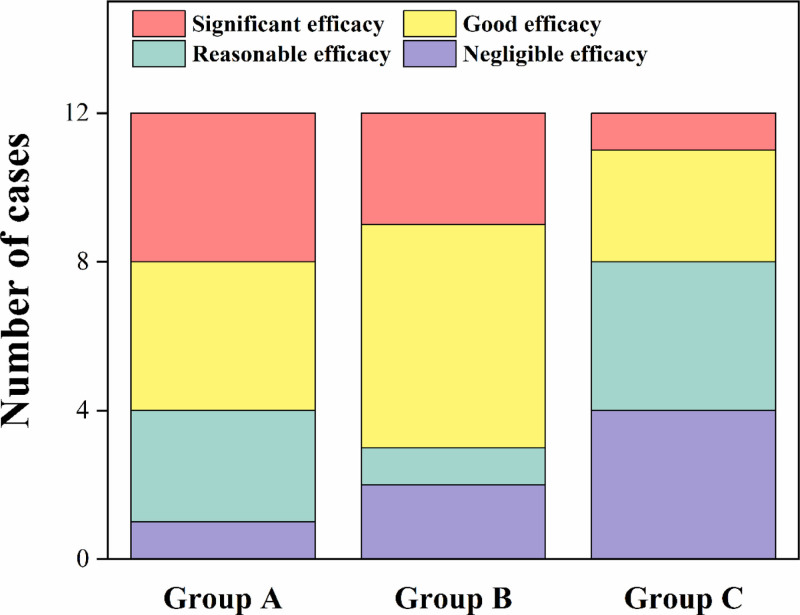
Number of cases with different overall efficacy levels (significant, good, reasonable, negligible) in Groups A, B and C.

The overall efficacy of hypnotics (Group A), hypnotics + OCSQA (Group B) and placebo (Group C) on sleep disorder of elderly were investigated. As shown in Table 9 and Figure 6, the ratios of significant and good efficacy cases were significantly higher in Groups A (67%) and B (75%) than in Group C (33%). More importantly, the ratio of significant and good efficacy cases in Group B was even higher than that in Group A.

**Table 9:** **Overall efficacy of Groups A, B and C** ($\bar{x}\pm \;s$**).**

Group	Number of cases	Number of significant efficacy cases	Number of good efficacy cases	Number of reasonable efficacy cases	Number of negligible efficacy cases
A	n = 12	4	4	3	1
B	n = 12	3	6	1	2
C	n = 12	1	3	4	4

### 3.6. Adverse events

Two of forty nine participants showed allergy to acupuncture and were excluded. One subject in Group B showed local congestion during the first intervention activity, but managed to complete the study. No other adverse events were reported during the study and 3 months after the study.

## 4. Discussion

### 4.1. Outcome of PSQI-based assessment

The relief of sleep disorder by OCSQA can be attributed to the release of endogenous analgesic substances (e.g., endorphins) facilitated by OCSQA^[37]^ and possible emotional support from the OCSQA physicians. The outcome of PSQI-based assessment revealed that the OCSQA can work synergistically with Estazolam as an adjuvant therapy to relieve sleep disorder of elderly.

### 4.2. Outcome of TCMS-based assessment

First, administration of hypnotics could effectively relieve frequent dreaming. Application of OCSQA had positive effects on relief of frequent dreaming and placebo had no significant influences on frequent dreaming. This may be attributed to the effects of OCSQA on human metabolism, especially for those with low exercise level (e.g., elderly).^[38]^

Then, administration of hypnotics could effectively reduce anxiety. Application of OCSQA had significant positive effects on anxiety relief and placebo had no significant influences on anxiety relief. This may be attributed to the influences of OCSQA on release of neurotransmitter and cortisol,^[39]^ as well as possible emotional support from the OCSQA physicians.

Meanwhile, administration of hypnotics could effectively prevent night sweating. Application of OCSQA had significant positive effects on prevention of night sweating and placebo had no significant influences on prevention of night sweating. This can be attributed to activation of the hypothalamic-pituitary-adrenal axis by OCSQA.^[40]^

Additionally, administration of hypnotics could effectively reduce susceptibility to influenza. Application of OCSQA had significant positive effects on prevention of susceptibility to influenza and placebo had no significant influences on susceptibility to influenza. This can be attributed to the contribution of OCSQA to immunity enhancement via regulation of metabolism.

### 4.3. Outcome of assessment based on serum levels of immunoglobulins

Treatment with hypnotics had significant effects on serum levels of IgG. Application of OCSQA led to enhanced efficacy of hypnotics from the perspective of IgG level in serum. Nevertheless, the influences of application of OCSQA on treatment with hypnotics can barely be reflected by the serum level of IgM.

### 4.4. Outcome of assessment based on overall efficacy

Treatment with hypnotics had significant efficacy on sleep disorder of elderly. Application of OCSQA as an adjuvant therapy can further enhance the efficacy of hypnotics in this case.

### 4.5. Risks of adverse events

Despite some minor adverse events, the proposed method showed low risks of adverse events during the study and 3 months after the study.

### 4.6. Limitations

Despite the results achieved, the present study exhibits several limitations: the ages of participants may affect the efficacy of different interventions, although no significant inter-group differences were present; the effects of race on the results were not taken into consideration as all participants enrolled in this study were Chinese; PSQI and TCMS are essentially subjective tools, and more objective tools such as blood test, sleep latency test and polysomnography shall be employed in future studies to consolidate the conclusions.

## 5. Conclusions

The effects of OCSQA as adjuvant therapy with hypnotic treatment on sleep disorder of elderly were explored. The results demonstrated that hypnotics could significantly relieve sleep disorder of elderly, as reflected by decreased PSQI, reduced TCMS scores, increased serum levels of immunoglobins and enhanced overall efficacy. OCSQA had positive impacts on such relief, as reflected by further improvements in most indicators; OCSQA can also relieve side-effects (e.g., anxiety, ataxia), as reflected by reduced PSQI and TCMS scores. Additionally, no significant adverse events were reported. Overall, OCSQA is an effective and safe adjuvant therapy for sleep disorder of elderly.

## Author contributions

**Conceptualization:** Ziping Luo.

**Formal analysis:** Ziping Luo.

**Funding acquisition:** Wenkun Luo.

**Investigation:** Qun Yi.

**Project administration:** Wenkun Luo.

**Resources:** Qun Yi.

## Supplementary Material


