# Supplementary figures and images for: Effects of opening and closing six-qi acupuncture as adjuvant therapy for sleep disorders of elderly
Source: Medicine (Baltimore). 2025 Aug 8;104(32):e43841. doi: 10.1097/MD.0000000000043841 (PMC12338216; doi:10.1097/MD.0000000000043841)

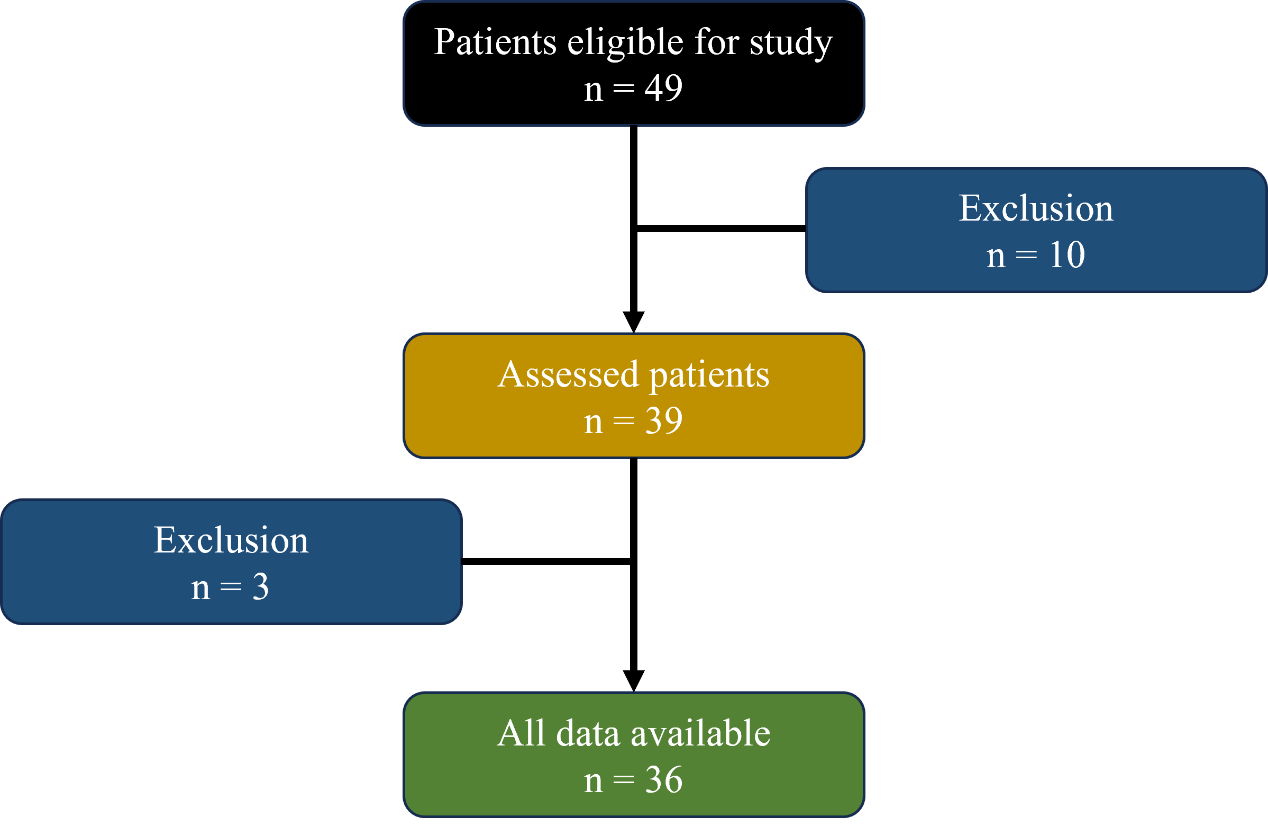


**Fig. S1** Flowchart of patient selection

Supplement: Supplementary file 1 [file medi-104-e43841-s001.docx]
