# Supplementary material for: Effects of opening and closing six-qi acupuncture as adjuvant therapy for sleep disorders of elderly
Source: Medicine (Baltimore). 2025 Aug 8;104(32):e43841. doi: 10.1097/MD.0000000000043841 (PMC12338216; doi:10.1097/MD.0000000000043841)
Supplement: Supplementary file 2 [file medi-104-e43841-s002.docx]

**Table S1** Demographic data of the participants enrolled in this study (1-12 = Group A, 13-24 = Group B, 25-36 = Group C)

| No. | Age (years) | Gender | Marital status | Fundamental diseases |
| --- | --- | --- | --- | --- |
| 1 | 65 | M | Married | Diabetes, hypertension, coronary heart disease |
| 2 | 64 | M | Divorced | Hypertension, coronary heart disease |
| 3 | 61 | M | Married | Diabetes, hypertension, asthma |
| 4 | 69 | F | Widowed | Diabetes, hypertension |
| 5 | 65 | M | Married | Diabetes, hypertension |
| 6 | 71 | M | Widowed | Diabetes, hypertension, Alzheimer’s disease |
| 7 | 62 | F | Married | Diabetes, asthma |
| 8 | 72 | F | Married | Diabetes |
| 9 | 64 | F | Married | Hypertension |
| 10 | 66 | M | Married | Diabetes, COPD |
| 11 | 76 | F | Married | Diabetes, COPD |
| 12 | 70 | M | Married | Diabetes, |
| 13 | 71 | M | Married | Diabetes, Alzheimer’s disease |
| 14 | 75 | F | Widowed | Hypertension, asthma |
| 15 | 68 | F | Married | / |
| 16 | 62 | M | Married | Hypertension, COPD |
| 17 | 60 | M | Married | COPD |
| 18 | 69 | F | Married | Hypertension, asthma |
| 19 | 75 | M | Married | Coronary heart disease |
| 20 | 76 | M | Married | Diabetes, coronary heart disease |
| 21 | 74 | F | Married | Diabetes |
| 22 | 71 | F | Married | Alzheimer’s disease |
| 23 | 66 | M | Divorced | Diabetes |
| 24 | 62 | M | Married | Hypertension |
| 25 | 72 | M | Married | Diabetes, Alzheimer’s disease |
| 26 | 70 | F | Married | Hypertension, asthma |
| 27 | 63 | M | Married | Diabetes, hypertension, Alzheimer’s disease |
| 28 | 61 | M | Married | Diabetes, hypertension |
| 29 | 62 | M | Married | Diabetes, hypertension, Alzheimer’s disease |
| 30 | 71 | F | Married | Hypertension |
| 31 | 73 | F | Married | COPD |
| 32 | 68 | F | Divorced | Diabetes, hypertension |
| 33 | 69 | M | Married | Coronary heart disease |
| 34 | 70 | M | Widowed | Hypertension, asthma |
| 35 | 67 | F | Married | Diabetes, hypertension |
| 36 | 71 | F | Widowed | Diabetes, hypertension, Alzheimer’s disease |

Name: Date:

**Pittsburgh Sleep Quality Index (PSQI)**

Instructions: The following questions relate to your usual sleep habits during the past month only. Your answers should indicate the most accurate reply for the majority of days and nights in the past month. **Please answer all questions.**

1. During the past month, what time have you usually gone to bed at night?
2. During the past month, how long (in minutes) has it usually taken you to fall asleep each night?
3. During the past month, what time have you usually gotten up in the morning?
4. During the past month, how many hours of actual sleep did you get at night? (This may be different than the number of hours you spent in bed.)

| 5. During the past month, how often have you had trouble sleeping because you… | Not during the past month | Less than once a week | Once or twice a week | Three or more times a week |
| --- | --- | --- | --- | --- |
| a. Cannot get to sleep within 30 minutes |  |  |  |  |
| b. Wake up in the middle of the night or early morning |  |  |  |  |
| c. Have to get up to use the bathroom |  |  |  |  |
| d. Cannot breathe comfortably |  |  |  |  |
| e. Cough or snore loudly |  |  |  |  |
| f. Feel too cold |  |  |  |  |
| g. Feel too hot |  |  |  |  |
| h. Have bad dreams |  |  |  |  |
| i. Have pain |  |  |  |  |
| j. Other reason(s), please describe: |  |  |  |  |
| 6. During the past month, how often have you taken medicine to help you sleep (prescribed or  “over the counter”)? |  |  |  |  |
| 7. During the past month, how often have you had trouble staying awake while driving, eating meals, or engaging in social activity? |  |  |  |  |
|  | No problem at all | Only a very slight problem | Somewhat of a problem | A very big problem |
| 8. During the past month, how much of a problem has it been for you to keep up enough enthusiasm to get things done? |  |  |  |  |
|  | Very good | Fairly good | Fairly bad | Very bad |
| 9. During the past month, how would you rate your sleep quality overall? |  |  |  |  |

|  | No bed partner or room mate | Partner/room mate in other room | Partner in same room but not same bed | Partner in same bed |
| --- | --- | --- | --- | --- |
| 10. Do you have a bed partner or room mate? |  |  |  |  |
|  | Not during  the past month | Less than once a week | Once or twice a week | Three or  more times a week |
| If you have a room mate or bed partner, ask him/her how often in the past month you have  had: |  |  |  |  |
| a. Loud snoring |  |  |  |  |
| b. Long pauses between breaths while asleep |  |  |  |  |
| c. Legs twitching or jerking while you sleep |  |  |  |  |
| d. Episodes of disorientation or confusion during sleep |  |  |  |  |
| e. Other restlessness while you sleep, please describe: |  |  |  |  |

**Scoring the PSQI**

The order of the PSQI items has been modified from the original order in order to fit the first 9 items (which are the only items that contribute to the total score) on a single page. Item 10, which is the second page of the scale, does not contribute to the PSQI score.

In scoring the PSQI, seven component scores are derived, each scored 0 (no difficulty) to 3 (severe difficulty). The component scores are summed to produce a global score (range 0 to 21). Higher scores indicate worse sleep quality.

**Component 1: Subjective sleep quality—question 9** Response to Q9 Component 1 score Very good 0

Fairly good 1

Fairly bad 2

Very bad 3

**Component 2: Sleep latency—questions 2 and 5a**

Component 1 score:

Response to Q2 Component 2/Q2 subscore

< 15 minutes 0

16-30 minutes 1

31-60 minutes 2

> 60 minutes 3

Response to Q5a Component 2/Q5a subscore Not during past month 0

Less than once a week 1

Once or twice a week 2

Three or more times a week 3

Sum of Q2 and Q5a subscores Component 2 score

0 0

1-2 1

3-4 2

5-6 3

**Component 3: Sleep duration—question 4**

Response to Q4 Component 3 score

> 7 hours 0

6-7 hours 1

5-6 hours 2

< 5 hours 3

**Component 4: Sleep efficiency—questions 1, 3, and 4**

Component 2 score:

Component 3 score:

Sleep efficiency = (# hours slept/# hours in bed) X 100% # hours slept—question 4

# hours in bed—calculated from responses to questions 1 and 3 Sleep efficiency Component 4 score

> 85% 0

75-84% 1

65-74% 2

< 65% 3

Component 4 score:

**Component 5: Sleep disturbance—questions 5b-5j** Questions 5b to 5j should be scored as follows: Not during past month 0

Less than once a week 1

Once or twice a week 2

Three or more times a week 3

Sum of 5b to 5j scores Component 5 score

0 0

1-9 1

10-18 2

19-27 3

Component 5 score:

**Component 6: Use of sleep medication—question 6**

Response to Q6 Component 6 score

Not during past month 0

Less than once a week 1

Once or twice a week 2

Three or more times a week 3

Component 6 score:

**Component 7: Daytime dysfunction—questions 7 and 8** Response to Q7 Component 7/Q7 subscore Not during past month 0

Less than once a week 1

Once or twice a week 2

Three or more times a week 3

Response to Q8 Component 7/Q8 subscore No problem at all 0

Only a very slight problem 1

Somewhat of a problem 2

A very big problem 3

Sum of Q7 and Q8 subscores Component 7 score

0 0

1-2 1

3-4 2

5-6 3

Component 7 score:

**Global PSQI Score:** Sum of seven component scores:

Copyright notice: The Pittsburgh Sleep Quality Index (PSQI) is copyrighted by Daniel J. Buysse, M.D. Permission has been granted to reproduce the scale on this website for clinicians to use in their practice and for researchers to use in non- industry studies. For other uses of the scale, the owner of the copyright should be contacted.

Citation: Buysse, DJ, Reynolds CF, Monk TH, Berman SR, Kupfer DJ: The Pittsburgh Sleep Quality Index (PSQI): A new instrument for psychiatric research and practice. Psychiatry Research 28:193-213, 1989
